# microRNA-4516 Contributes to Different Functions of Epithelial Permeability Barrier by Targeting Poliovirus Receptor Related Protein 1 in Enterovirus 71 and Coxsackievirus A16 Infections

**DOI:** 10.3389/fcimb.2018.00110

**Published:** 2018-04-09

**Authors:** Yajie Hu, Jie Song, Longding Liu, Ying Zhang, Lichun Wang, Qihan Li

**Affiliations:** Institute of Medical Biology, Chinese Academy of Medical Science and Peking Union Medical College, Yunnan Key Laboratory of Vaccine Research and Development on Severe Infectious Diseases, Kunming, China

**Keywords:** enterovirus 71 (EV-A71), coxsackievirus A16 (CV-A16), hand Foot and mouth disease (HFMD), microRNA-4516 (miR-4516), poliovirus receptor related protein 1 (PVRL1), Airway epithelial barrier

## Abstract

Enterovirus 71 (EV-A71) and coxsackievirus A16 (CV-A16) remain the predominant etiological agents of hand, foot, and mouth disease (HFMD). The clinical manifestations caused by the two viruses are obviously different. CV-A16 usually triggers a repeated infection, and airway epithelial integrity is often the potential causative factor of respiratory repeated infections. Our previous studies have demonstrated that there were some differentially expressed miRNAs involved in the regulation of adhesion function of epithelial barrier in EV-A71 and CV-A16 infections. In this study, we compared the differences between EV-A71 and CV-A16 infections on the airway epithelial barrier function in human bronchial epithelial (16HBE) cells and further screened the key miRNA which leaded to the formation of these differences. Our results showed that more rapid proliferation, more serious destruction of 16HBE cells permeability, more apoptosis and disruption of intercellular adhesion-associated molecules were found in CV-A16 infection as compared to EV-A71 infection. Furthermore, we also identified that microRNA-4516 (miR-4516), which presented down-regulation in EV-A71 infection and up-regulation in CV-A16 infection was an important regulator of intercellular junctions by targeting Poliovirus receptor related protein 1(PVRL1). The expressions of PVRL1, claudin4, ZO-1 and E-cadherin in CV-A16-infected cells were significantly less than those in EV-A71-infected cells, while the expressions of these proteins were subverted when pre-treated with miR-4516-overexpression plasmid in EV-A71 infected and miR-4516-knockdown plasmid in CV-A16 infected 16HBE cells. Thus, these data suggested that the opposite expression of miR-4516 in EV-A71 and CV-A16 infections might be the initial steps leading to different epithelial impairments of 16HBE cells by destroying intercellular adhesion, which finally resulted in different outcomes of EV-A71 and CV-A16 infections.

## Introduction

Hand, foot, and mouth disease (HFMD) is a highly contagious disease that has mainly circulated in the Asia-Pacific region over the past several years and causes significant morbidity and mortality, particularly in infants and young children. Therefore, HFMD has become a serious public health problem in these areas (Aswathyraj et al., 2016). Enterovirus 71 (EV-A71) and coxsackievirus A16 (CV-A16) are both single-stranded, positive-sense RNA viruses that belong to human enterovirus species A of the genus *Enterovirus* within the *Picornaviridae* family and are the predominant etiological agents of sporadic and epidemic HFMD (Wang et al., 2013; Lin and Shih, 2014). Although EV-A71 and CV-A16 share approximately 80% sequence similarity, the clinical manifestations caused by the two viruses are obviously different (Lee et al., 2011). While patients infected with EV-A71 may easily progress to severe complications involving the central nervous system (CNS) such as meningitis, encephalitis and acute flaccid paralysis, patients infected with CV-A16 usually show mild, self-limiting disease symptoms (Cai et al., 2014; Sun et al., 2014). However, the underlying mechanisms leading to these differences have not yet been elucidated. Airway epithelial cells, positioned at the interface between the host and the external environment, constitute a physical barrier via their tightly regulated cell-cell interactions which can form adhesive forces that connect neighboring cells to efficiently fight the entry of pathogens most of the time (Vareille et al., 2011). Paradoxically, these cells are also a major portal for the entry of microbial infections, mainly due to many pathogens have evolved a series of strategies to cross the airway epithelial barrier, which eventually causes severe respiratory infectious disease (Vareille et al., 2011; Mateo et al., 2015) For example, rhinoviruses are able to impair epithelial integrity by interfering with the zonula occludens (ZO)-1 protein and then successfully invade host cells (Sajjan et al., 2008; Bhowmik et al., 2009). Currently, the postulated route of EV-A71 and CV-A16 transmission is primarily the fecal-oral route because both viruses can survive in the acidic pH of the stomach, enter the host through the intestines and spread to other regions of the body via the circulatory system (Lui et al., 2013). Nevertheless, EV-A71 and CV-A16 transmission via respiratory droplets has been reported (Wang et al., 2011). Moreover, our previous study also confirmed that EV-A71 infection presented more typical pathologic changes in the respiratory tract than those in the alimentary tract (Zhao et al., 2017). Thus, we speculate that variations in pathogenesis induced by EV-A71 and CV-A16 infections might be originally derived from different epithelial alterations.

microRNA (miRNA), as central regulators of eukaryotic gene expression, has been revealed to be involved in the infectious cycle of EV-A71 or CV-A16 (Ho et al., 2016). We also detected miRNA profiling in human bronchial epithelial (16HBE) cells following EV-A71 and CV-A16 infections by high-throughput sequencing and found that those oppositely expressed miRNAs in response to EV-A71 and CV-A16 infections might participate in modulating the function of the epithelial barrier (Hu et al., 2017). Furthermore, increasing evidence indicated that defects in cell-cell interactions could result in structural and functional abnormalities in the airway epithelial barrier, which could ultimately lead to recurrent infections, as seen in patients with cystic fibrosis (Livraghi and Randell, 2007), chronic obstructive pulmonary disease (COPD), and ciliary dyskinesis (Boon et al., 2014). Thus, it was hypothesized that differentially expressed miRNAs generated in the 16HBE cells with EV-A71 or CV-A16 infections might drive different epithelial barrier changes that could contribute to the situation of differentially clinical repeated infections.

## Materials and methods

### Cells and viruses

16HBE which was a human bronchial epithelial cell line was purchased from Jennino Biological Technology (Guangzhou, China) were propagated in Dulbecco's modified Eagle medium (DMEM) (Corning, USA) supplemented with L-glutamine, penicillin, streptomycin and 10% fetal bovine serum (FBS, Gibco, USA) at 37°C in a humidified atmosphere containing 5% CO_2_. EV-A71 (sub-genotype C4, GenBank: EU812515.1) or CV-A16-G20 strain (sub-genotype B, GenBank: JN590244.1) were, respectively, isolated from an epidemic in Fuyang, China, in 2008 and from an HFMD patient in Guangxi, China, in 2010 and were used throughout the study. 16HBE cells were grown in 6-well plates to 80% confluence and then infected with EV-A71 or CV-A16 at a multiplicity of infection (MOI) of 10. The infected cells were collected at 0, 6, and 12 h post infection (hpi). Cells infected with EV-A71 and CV-A16 for 0 hpi were used as controls.

African green monkey kidney (Vero) cells for viral plaque assays obtained from Institute of Medical Biology (Kunming, China) were cultivated in minimum essential medium (MEM) with 10% newborn bovine serum (NBS, Gibco, USA), L-glutamine and 1% penicillin/streptomycin under 37°C in a humidified atmosphere containing 5% CO_2_.

293T cells for dual-luciferase reporter assay provided by Institute of Medical Biology (Kunming, China) were cultured at 37°C, 5% CO_2_ incubator in DMEM plus 10% FBS, penicillin and streptomycin.

### Quantitative RT-PCR (qRT-PCR) for viral load

Total RNA was extracted from all samples using the standard TRIzol reagent (TIANGEN, China) protocol. The regions of standards generated from EV-A71-VP1 and CV-A16-VP1 RNA were amplified by the PrimeSTAR® HS reagent (TAKARA, Japan) with the primers shown in Table S1. The target 624 bp region (EV-A71-VP1) and the 682 bp region (CV-A16-VP1) were cloned into the pGM-T vector (TIANGEN, China) following the manufacturer's guidelines. A single recombinant clone containing the insert under the T7 promoter was selected, and the insert was transcribed *in vitro* using the TranscriptAid T7 High Yield Transcription kit (Thermo Scientific, USA) according to the manufacturer's recommendations. The *in vitro*-transcribed RNA was purified using the GeneJET RNA Purification kit (Thermo Scientific, USA) and quantified spectrophotometrically using the Nanodrop 2000 (Thermo Scientific, USA). The qRT-PCR process was performed using a One Step PrimeScript™ RT-PCR kit (TAKARA, Japan). To generate a standard curve for cycle thresholds (Cts) vs. virus copy number, the purified RNA was serially diluted to known concentrations in a range of 10^1^-10^8^ copies/μl and assayed concurrently with the test samples. The PCR conditions were as follows: 5 min at 42°C for reverse transcription and 10 s of denaturation at 95°C, followed by 95°C for 5 s and 60°C for 34 s repeated for 40 cycles. The reactions were run on a 7500 Fast Real-time PCR system (Applied Biosystems, USA). The qRT-PCR primers used for the EV-A71 and CV-A16 copy numbers are listed in Table S1. Each sample was tested in triplicate.

### Plaque assay

Cells and culture supernatants were collected together at 0, 6, and 12 hpi to assess the production of infectious viral particles. Then, cells were disrupted by 3 freeze-thaw cycles, and the virus titer was determined by a standard plaque assay. First, Vero cells were grown to a monolayer on 6-well plates and then inoculated with serial 10-fold dilutions of the thawed samples (1 ml per well). After 3 h of incubation to allow virus attachment, the wells were gently washed with phosphate-buffered saline (PBS), covered with media containing 1% agarose and placed into a 37°C CO_2_ incubator for 48 h. Next, the cells were fixed with 2 ml of 4% paraformaldehyde (PFA) (Solarbio, China) and incubated for 30 min at room temperature, and the 1% agarose was removed. The monolayer of cells was stained with a crystal violet staining solution for 15 min, and washed with ddH_2_O. Finally, visible plaques were counted by the naked eye and the plaque-forming units (pfu/ml) were calculated with the virus titer formula, where virus titer equals the number of plaques × (1 ml) × dilution factor. All samples were tested in triplicate.

### VP1 staining and flow cytometric analysis

16HBE cells were added to 6-well plates and infected with EV-A71 or CV-A16. After treatment, cells were harvested, washed once with PBS and then fixed and permeabilized according to the BD Cytofix/Cytoperm™ Kit (BD Biosciences, USA) recommendations. Subsequently, cells were stained with EV-A71/CV-A16-VP1 (1:100, Millipore, USA) for 30 min at room temperature, and washed twice with PBS. Secondary antibody containing FITC marker was added, and the plate was incubated for 30 min in the dark at room temperature. After incubation, cells were resuspended in 0.5 ml of PBS and assayed by a FACScan flow cytometer (BD Biosciences, USA). Each sample contained 10,000 cells, and three independent replicates of each sample were tested. FlowJo 7.6 software was used for data analysis.

### Measurement of 16HBE cells permeability

16HBE cells were cultured on transwell inserts placed in a 24-well plate until 100% confluence was reached; then, they were infected with EV-A71 or CV-A16. Following completion of the treatment, the permeability of cell monolayers was determined with an *in vitro* Vascular Permeability Assay kit (Millipore, USA). Briefly, the medium was carefully removed from the transwell insert, and 150 μl of FITC-Dextran working solution was added to the apical surface of 16HBE cells. The 24-well plate was protected from light and incubated for 20 min at room temperature. Next, the medium from each well of the receiver tray was thoroughly mixed, and 100 μl was transferred to the wells of a black 96-well opaque plate for fluorescence measurement. The fluorescein dye was measured in a multi-mode reader (Biotek, USA) at 480/530 nm (excitation/emission). The data are representative of at least three experiments.

### Apoptosis assay

The Annexin V/FITC and propidium iodide (PI) apoptosis detection kit (4A Biotech, China) was used to quantitatively measure the phosphatidylserine in apoptotic cells. The 16HBE cells were harvested at 0, 6, and 12 hpi and washed three times with ice-cold PBS. After washing, each sample was centrifuged at 1,500 rpm for 5 min at 4°C. Subsequently, 250 μl of buffer, 5 μl of Annexin V/FITC, and 10 μl of PI were added according to the manufacturer's instructions. The stained cells were immediately assayed by a FACScan flow cytometer and the data were analyzed by FlowJo 7.6 software.

### Detection of adhesion-related molecules and miR-4516 expressions by qRT-PCR

The total RNA from each sample was obtained and then subjected to qRT-PCR for all adhesion-related genes and miR-4516 by a One-Step SYBR® PrimeScript™ PLUS RT-PCR Kit (TAKARA, Japan) as previously described. U6 snRNA and GAPDH are served as an appropriate calibrator housekeeping gene for miR-4516 and adhesion-related genes quantitative analysis, respectively. The relative expression levels of these genes were calculated from the equation 2^−−ΔΔCt^. The qRT-PCR primers used in this analysis are given in Table S1. Each sample was tested in triplicates.

### Bioinformatic analysis

Based on the previous analysis on the miRNAs profiles in 16HBE cells infected by EV-A71 and CV-A16, we mainly focused on the 24 differentially expressed miRNAs involved in biological adhesion, cell adhesion and cell-cell adhesion. To further screen the key miRNAs, we then looked for intersections of miRNAs related to the above mentioned three GOs. Moreover, we constructed a miRNA-targets network according to the interaction between the chosen miRNA and targets. According to the targets sequences, we also predicted the protein structure for further avoiding false positives by computational methods.

### Dual-luciferase reporter assay

miRNA-negative control (NC) plasmid, miR-4516-overexpression plasmid, 3′-UTR NC plasmid, PVRL1 3′-UTR plasmid, PVRL1 3′UTR-Mutant plasmid and TRAF6 3′-UTR plasmid (which was demonstrated that it was a direct target of miR-4516) used in this study were synthesized by the Shanghai Genechem Co., LTD. 293T cells were plated into 24-well plate at a density of 1 × 10^5^ cells/well. Once cells reached approximately 80% confluence, co-transfection, including miRNA-NC plasmid or miR-4516- overexpression plasmid with the other four plasmids, respectively, was carried out using the FuGENE® HD Transfection Reagent (Promega, USA) according to the manufacturer's instructions. 48 h after co-transfection, luciferase and renilla signals were measured using the Dual Luciferase Reporter Assay Kit (Promega, USA) according to the manufacturer's instructions and normalized against the activity of the Renilla/firefly luciferase gene. All transfection assays were tested in three independent biological replicates.

Additionally, the regulation mechanism of miR-4516 on PVRL1 was further detected by WB in 293T cells subjected to miR-4516-up transfection. The detailed experimental protocols of WB are described below.

### Immunofluorescence (IF) microscopy

16HBE cells were seeded onto poly-L-lysine-coated coverslips (Solarbio, China) and treated as previously described. At the indicated time, the cells were washed in PBS, fixed with 4% PFA (Solarbio, China) and permeabilized with 1% Triton X-100 in PBS. The cells were blocked with 5% BSA at room temperature for 1 h and then incubated with the primary antibodies in blocking solution against EV-A71/CV-A16-VP1 (1:1,000, Millipore, USA), Nectin1 (1:100, Novusbio, USA), Claudin4 (1:200, Abcam, USA), E-cadherin (1:500, Abcam, USA) and ZO-1 (1:200, Abcam, USA) overnight at 4°C. Next, cells were washed with PBS three times and then incubated with Alexa Fluor 594-conjugated donkey anti-rabbit IgG (Abcam, USA), Alexa Fluor 647-conjugated donkey anti-mouse IgG (Millipore, USA) or Alexa Fluor 488-conjugated donkey anti-rabbit IgG (Biolegend, USA) for 1 h at room temperature. The nuclei were counterstained with 4′, 6-diamidino-2-phenylindole (DAPI, 1:4,000, Beyotime, China). Slides were mounted with antifade reagent (Solarbio, China) and observed using a laser-scanning confocal microscope (Leica, Germany). The images were captured and processed using Adobe Photoshop 7.0 software.

### WB detection

Three independent samples from each group were harvested and homogenized, and the cells were lysed in RIPA buffer containing a protease inhibitor mixture (Beyotime, China) for 30 min on ice. The cell lysates were centrifuged at 12,000 rpm for 10 min at 4°C. After centrifugation, the insoluble material was removed, and the total protein concentration in supernatant was measured with a BCA protein assay kit (Beyotime, China). Equal amounts of protein (30 μg) were loaded on 8–15% sodium dodecyl sulfate-polyacrylamide gels for electrophoresis (SDS-PAGE), electrophoretically separated and transferred to polyvinylidene difluoride (PVDF) membranes. The membranes were blocked for 1 h at room temperature in 5% non-fat dry milk in Tris-buffered saline with Tween-20 (TBST) and then incubated overnight with the primary antibody, which included antibodies against EV-A71 (1:1,000, Millipore, USA), Nectin1 (1:1,000, Novusbio, USA), Claudin4 (1:1,000, Abcam, USA), E-cadherin (1:2,000, Abcam, USA), ZO-1 (1:150, Abcam, USA), and GAPDH (1:500, as loading control, Boster, China). After extensive washing with TBST, the membranes were incubated with species-specific horseradish peroxidase-conjugated secondary antibodies (1:12,000, Abmart, USA) for 1 h at room temperature. Immunoreactive bands were developed with an enhanced chemiluminescence kit (Beyotime, China) following the manufacturer's manual and then detected with a ChemiDoc™ Imaging System (Bio-Rad, USA). All gray values of the protein bands were quantified using Image J, and the values represent the relative immunoreactivity of each protein normalized to the respective loading control.

### Statistical analysis

Statistical analysis was carried out using SPSS 18.0 software (IBM SPSS, USA). All data are expressed as the mean ± the standard error of the mean (SEM). Comparisons between two groups were analyzed using an unpaired, two-tailed Student's *t*-test. Multiple groups were compared using one-way analysis of variance (ANOVA) with a Bonferroni *post hoc* test. A *P* value of less than 0.05 was considered statistically significant.

## Results

### EV-A71 and CV-A16 replicate in 16HBE cells

**Figure 1:**
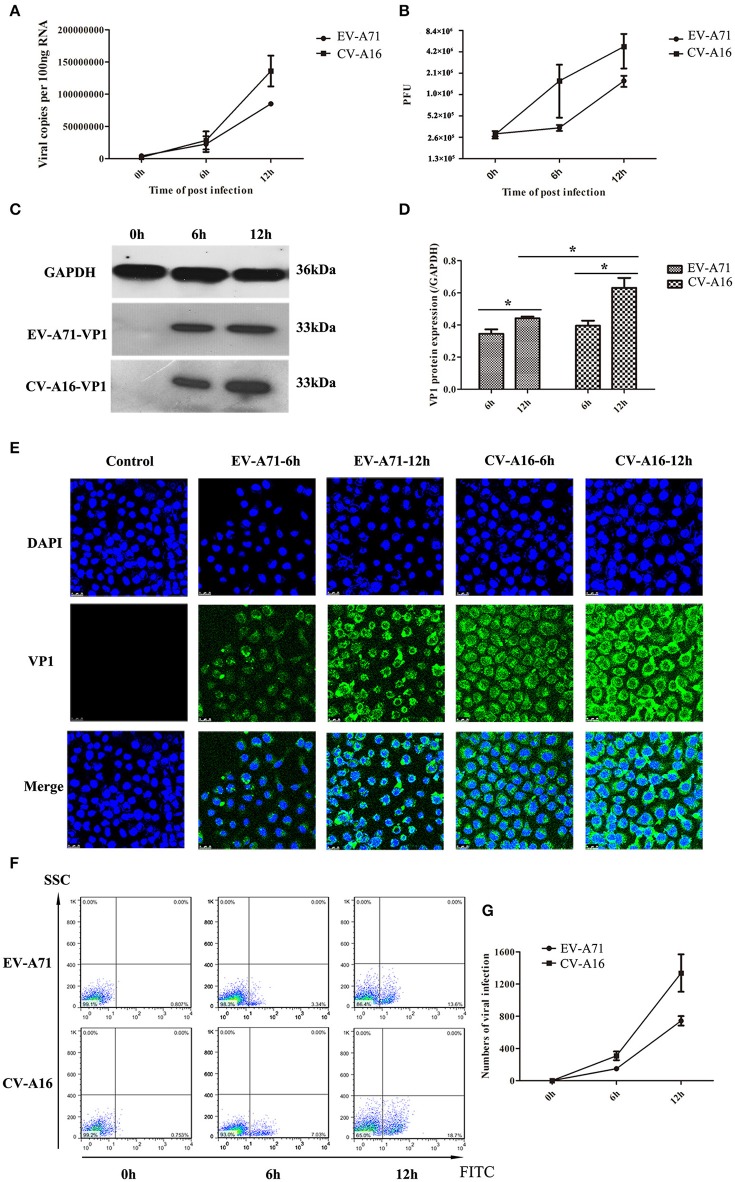
Viral growth curves and infection rates of EV-A71 and CV-A16 in 16HBE cells at an MOI of 10. **(A)** The replication kinetics of EV-A71 and CV-A16 were determined by qRT-PCR. **(B)** Infectious virus particles from EV-A71- and CV-A16-infected cells were quantitated by a traditional plaque assay. **(C)** Expression of EV-A71/CV-A16-VP1 protein in 16HBE cells subjected to EV-A71 and CV-A16. **(D)** The statistical results of EV-A71/CV-A16-VP1 protein expression displayed as a histogram. Significant differences among these groups are indicated by ^*^P < 0.05. **(E)** The efficacy of EV-A71 and CV-A16 infections was measured by an immunofluorescence assay over a period of 12 h. **(F)** The rate of EV-A71 and CV-A16 infection was evaluated quantitatively by flow cytometry. **(G)** The data of flow cytometry analysis represented as a line chart. In **(A,B,D,G)** the results are the averages of triplicate samples. Error bars represent the standard errors of the mean (SEM).

It has been previously shown that EV-A71 and CV-A16 could infect infant rhesus monkeys through the respiratory tract (Wang et al., 2011). To determine whether the airway epithelial cells are the key portal for EV-A71 and CV-A16 entry, replication kinetics studies were carried out in 16HBE cells. In EV-A71- and CV-A16-infected cells, the amplification of viral RNA and the production of infectious virus particles showed a constant rise (Figures 1A,B) with time, suggesting that the 16HBE cells were highly susceptible to EV-A71 and CV-A16. Nonetheless, the amplification of the viral RNA and the production of infectious virus particles in EV-A71 infection were not as high as those in CV-A16 infection at all-time points, indicating that the infective capacity of CV-A16 was stronger than that of EV-A71. To further address the question of whether CV-A16 possesses a more efficient replication ability to infect the epithelium, WB and IF experiments were employed. The results shown in Figures 1C,D revealed that although the expression of the viral structural protein VP1 of EV-A71 and CV-A16 significantly increased with time, cells infected with CV-A16 had remarkably higher VP1 protein levels as compared to cells infected with EV-A71 at 12 hpi (*P* < 0.05). Furthermore, we also observed that the viral accumulation in VP1-positive cells followed by CV-A16 infection notably exceeded that in EV-A71 infection (Figure 1E). Finally, the rate of EV-A71 and CV-A16 infections was evaluated quantitatively by flow cytometry and we found that the rate of CV-A16 infection was higher than that of EV-A71 infection with time (Figures 1F,G). Collectively, these results suggested that CV-A16 possessed a faster replication capacity in 16HBE cells than EV-A71.

### EV-A71 and CV-A16 infections enhance 16HBE cells permeability

**Figure 2:**
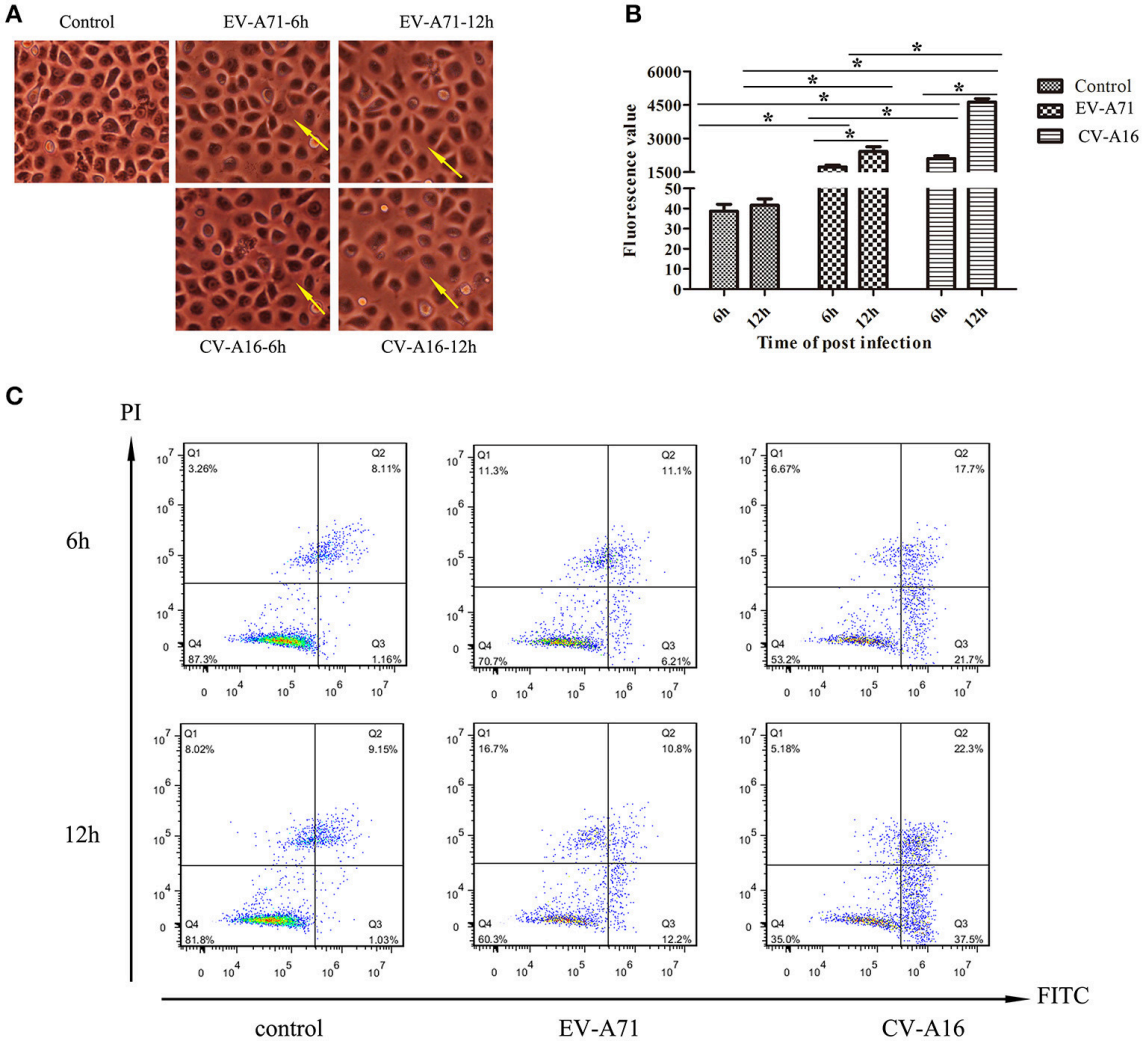
Increased epithelial permeability due to EV-A71 and CV-A16 infections in 16HBE cells. **(A)** Morphological changes of 16HBE cells affected by EV-A71 and CV-A16 infections at 6 and 12 hpi. The yellow arrow indicates the enlarged intercellular gaps. **(B)** Epithelial permeability was detected by a paracellular FITC-dextran flux assay. Significant differences among these groups are indicated by ^*^P < 0.05. **(C)** EV-A71 and CV-A16 infections promote 16HBE cell apoptosis, including early apoptotic and late apoptotic.

To evaluate the effects of EV-A71 and CV-A16 infections on epithelial permeability, we observed the morphological changes of 16HBE cells with an optical microscope and performed a paracellular FITC-dextran flux assay by monitoring the ability of FITC-dextran to migrate from the upper compartment to the bottom compartment. The results displayed that the cellular morphology of 16HBE cells in control group was intact, whereas intercellular gaps became gradually enlarged in 16HBE cells with the progression of EV-A71 and CV-A16 infections, especially CV-A16 infection (Figure 2A). Furthermore, there were dramatic differences in epithelial permeability changes between infected and control cells at 6 and 12 hpi (*P* < 0.05, Figure 2B). The integrity of epithelial barrier was compromised in response to EV-A71 and CV-A16 infections. Additionally, it was found that CV-A16 infection could result in more permeable than EV-A71 infection at 6 and 12 hpi in 16HBE cells (*P* < 0.05).

### EV-A71 and CV-A16 infections induce apoptosis in 16HBE cells

Apoptotic responses in virally infected cells are key protective mechanisms to prevent and minimize the spread of a virus, but extensive apoptosis causes tissue damage (Koyama et al., 2003). Hence, we next explored whether the disruption of the epithelial barrier was partly due to the increasing apoptosis in EV-A71 and CV-A16 infections. A flow cytometry analysis determined that EV-A71 caused early apoptosis accounted for 6.21 and 12.2% of the cells at 6 and 12 hpi, respectively, while CV-A16 led to early apoptosis accounted for 21.7 and 37.5% of the cells at 6 and 12 hpi, respectively. Moreover, the proportion of late apoptosis or necrosis was, respectively, 11.1 and 10.8% in the EV-A71 infection at 6 and 12 hpi and 17.7 and 23.3% in the CV-A16 infection at 6 and 12 hpi (Figure 2C). Thus, this result revealed that CV-A16 infection could promote more apoptosis as compared to EV-A71 infection.

### Different alterations in adhesion-related molecules in 16HBE cells followed EV-A71 and CV-A16 infections

**Figure 3:**
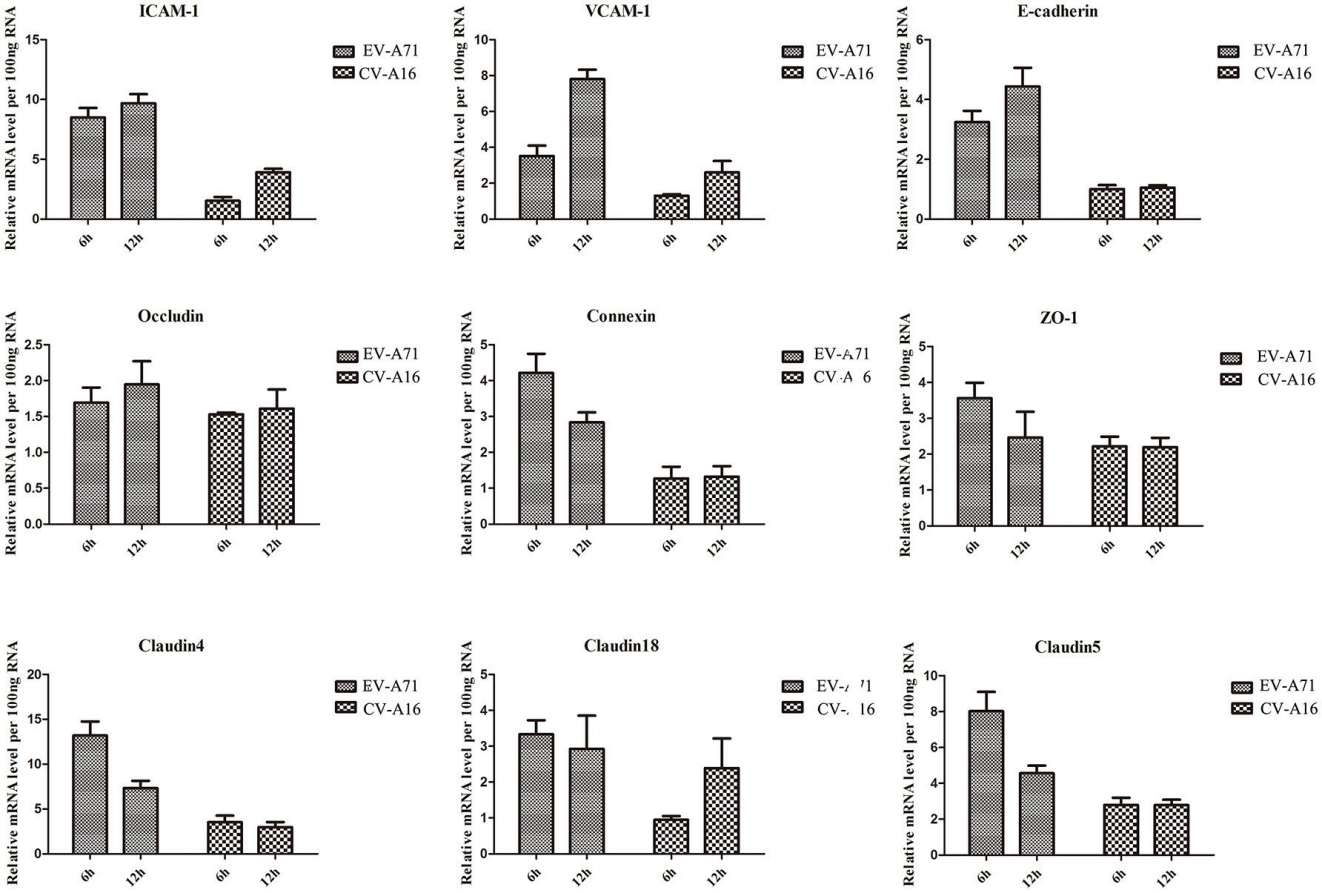
Measurement by qRT-PCR of adhesion-related molecules in 16HBE cells following EV-A71 and CV-A16 infections. The expression levels of adhesion-related genes mRNA were normalized to the constitutive expression level of GAPDH mRNA. Data in all panels are representative of at least 3 separate experiments.

Airway epithelial cells possess cell-cell junction molecules to build up a solid physical barrier. Hence, we identified the mRNA expression level of the adhesion-associated molecules by qRT-PCR. The results showed that all of the cellular adhesion-related molecules, including immunoglobulin superfamily molecules (e.g., ICAM, VCAM), gap junction (GJ) molecule (e.g., connexin), tight junctions (TJs) molecule (e.g., occludin, ZO-1), and Ca^2+^ dependent cell adhesion molecule family (Cadherin) molecules (e.g., E-cadherin, cadherin4/18/5), were notably down-regulated in CV-A16 infection compared with EV-A71 infection (Figure 3). Thus, these data further indicated that CV-A16 infection easily led to a disruption of cell-cell junction as compared to EV-A71 infection.

### PVRL1-3′UTR is the direct target of miR-4516 which was the key miRNA involved in regulation of epithelial barrier function

**Figure 4:**
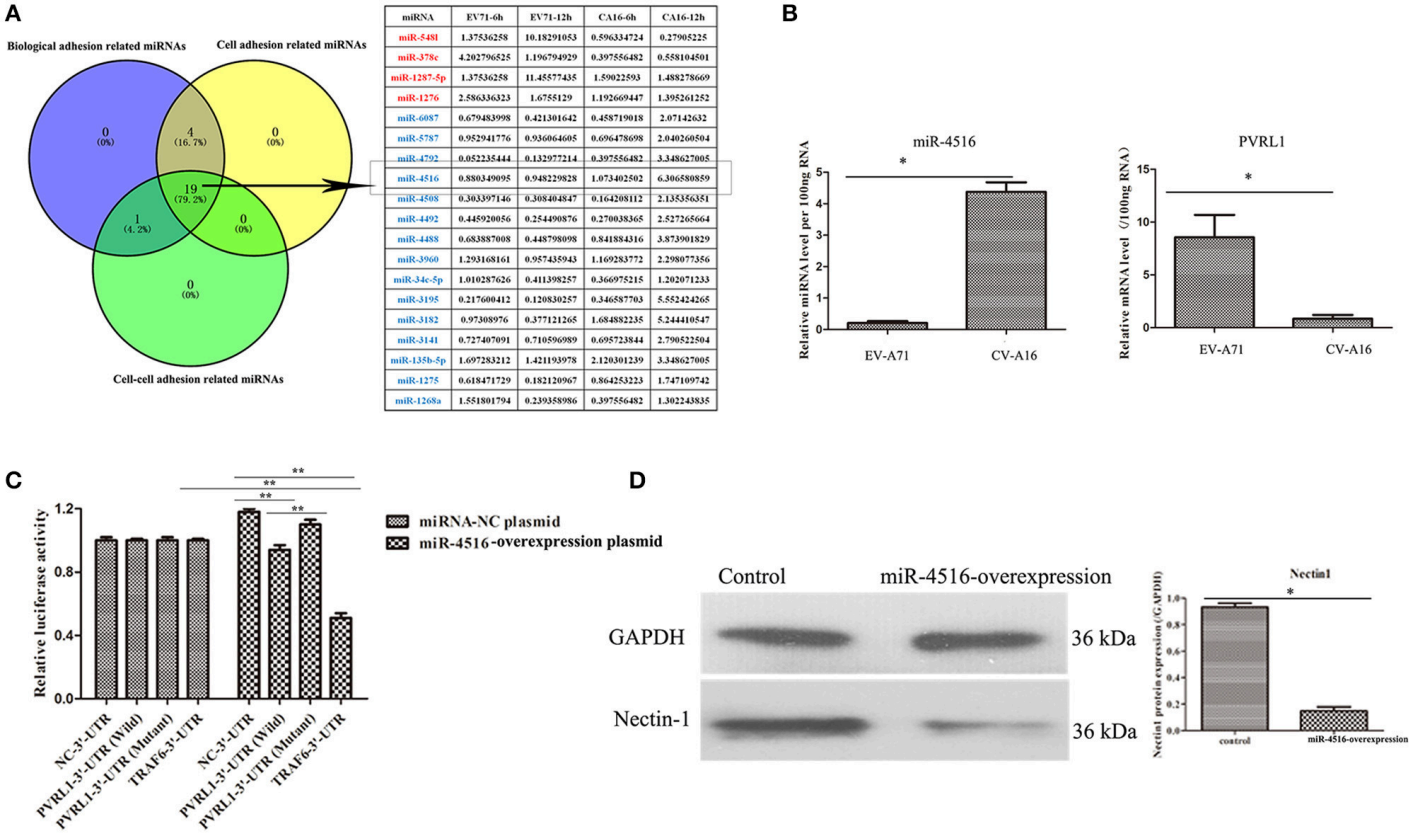
PVRL1 was speculated as the direct target of miR-4516. **(A)** The only miRNAs identified by the three adhesion-related GOs were considered as key miRNAs for in-depth research. The miRNAs highlighted in red and blue represent the up-regulation and down-regulation in the EV-A71-infected groups, respectively, indicating adverse regulation in the CV-A16-infected groups, respectively. The miR-4516 was chosen for further study due to its most significant change. **(B)** miR-4516 and PVRL1 expression were detected by qRT-PCR array in 16HBE cells with EV-A71 and CV-A16 infection. **(C)** The target interaction between miR-4516 and PVRL1 was determined by a dual-luciferase reporter assay. Significant differences among these groups are indicated by ^**^P < 0.01 and ^*^P < 0.5. **(D)** WB analysis of PVRL1 expression in transfected cells. Transfection with the miR-4516-overexpression plasmid resulted in a significant reduction of PVRL1 protein expression in 16HBE cells. GAPDH was used as a reference.

We previously reported that biological adhesion-, cell adhesion- and cell-cell adhesion-related miRNAs are the predominate miRNAs involved in modulating the function of the epithelial barrier (Hu et al., 2017). Among these miRNAs, miR-4516, which was down-regulated in EV-A71 infection and up-regulated in CV-A16 infection, presented the most significant change (Figure 4A). Furthermore, the expression level of PVRL1, a predicted target of miR-4516, was remarkably increased in EV-A71 infection and markedly decreased in CV-A16 infection (Figure 4B). Subsequently, the data of dual-luciferase reporter assay presented that there were no significant differences in relative luciferase activity when co-transfecting miRNA-NC plasmid with 3′-UTR NC plasmid, PVRL1 3′-UTR plasmid, PVRL1 3′UTR-Mutant plasmid, TRAF6 3′-UTR plasmid, but the relative luciferase activity was apparently decreased in miR-4516+PVRL1 3′-UTR group (*P* = 0.000434958) and miR-4516+TRAF6 3′-UTR group (which was a positive control in this experiment, *P* = 9.88497E-06) as compared to miR-4516+3′-UTR NC group (Figure 4C). Moreover, the relative luciferase activity was obviously increased in miR-4516+ PVRL1 3′-UTR Mutant group (*P* = 0.003875413) compared with miR-4516+ PVRL1 3′-UTR (Figure 4C). Therefore, it was confirmed that PVRL1 3′-UTR was the direct target of miR-4516. Finally, based on the cellular mechanisms of miRNAs, which is the regulation of gene expression by the sequence-selective targeting of mRNA leading to their cleavage or a reduction in translational efficiency, we checked the protein expression level of PVRL1 by a WB assay and also found that PVRL1 protein expression was sharply declined in 293T cells transfected with the miR-4516-up plasmid (Figure 4D).

### miR-4516 destroys the location of junctional proteins and decreases the expression levels of junctional proteins

**Figure 5:**
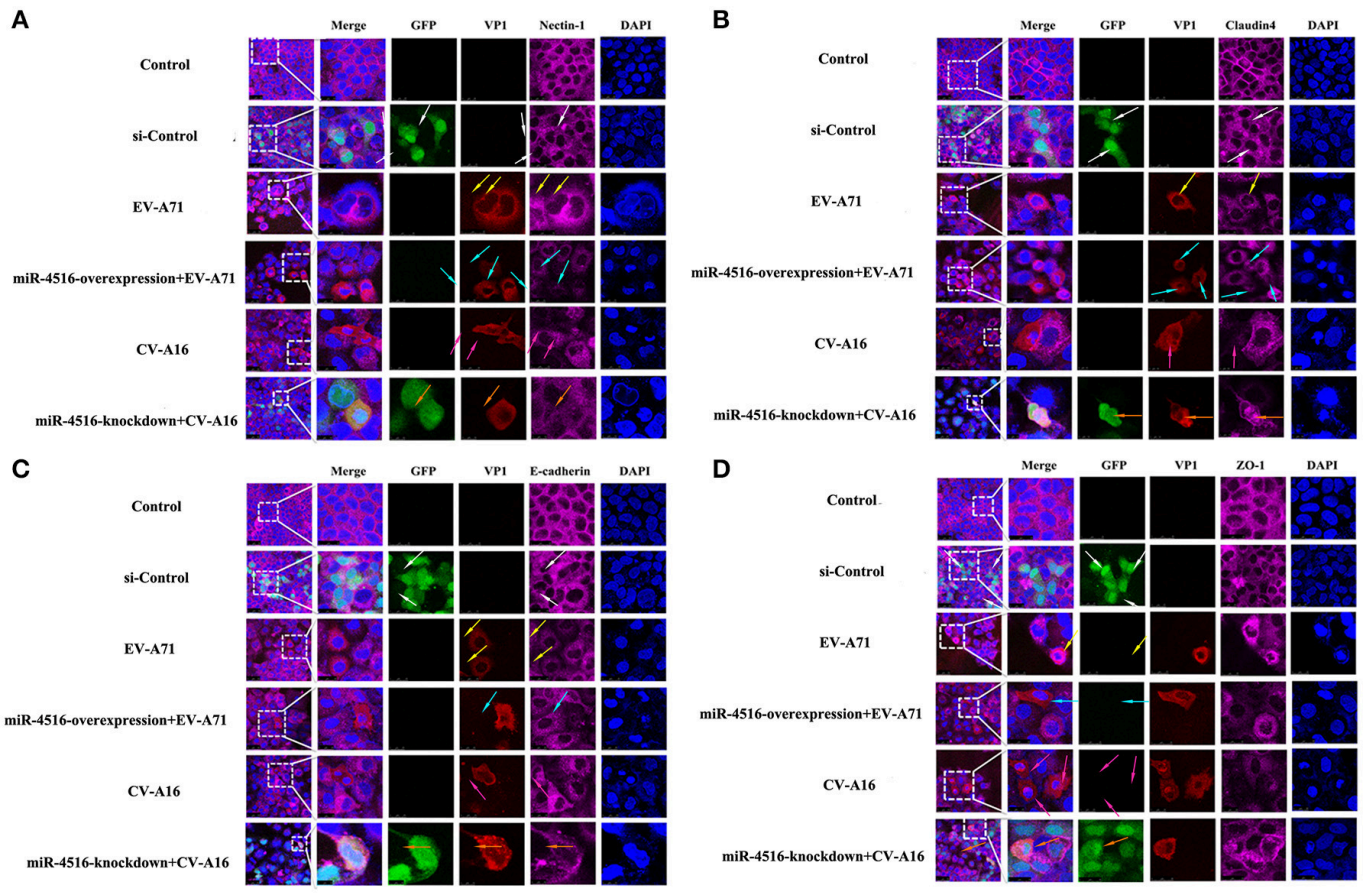
Confocal imaging showed the junctional protein localization of nectin1 **(A)**, claudin4 **(B)**, E-cadherin **(C)**, and ZO-1 **(D)** in differently treated 16HBE cells. The si-control and miR-4516-knockdown plasmids contain a GFP tag (green color). EV-A71/CV-A16-VP1, junctional proteins and the cell nucleus are indicated by red, purple and mazarine, respectively. The white arrows indicate that the transfected cells with si-control still show a normal “chicken wire” appearance of the above junctional proteins. The yellow arrows indicate that the EV-A71-infected cells displayed slighter changes of these proteins. The light blue arrows represent that the EV-A71-infected cells with miR-4516-overexpression plasmid pretreatment showed remarkably lower levels of these proteins. The rose red arrows denote that the CV-A16-infected cells also show a large reduction of these proteins. The saffron arrows indicate that CV-A16-infected cells pre-treated with the miR-4516-knockdown plasmid show less recovery of these proteins.

**Figure 6:**
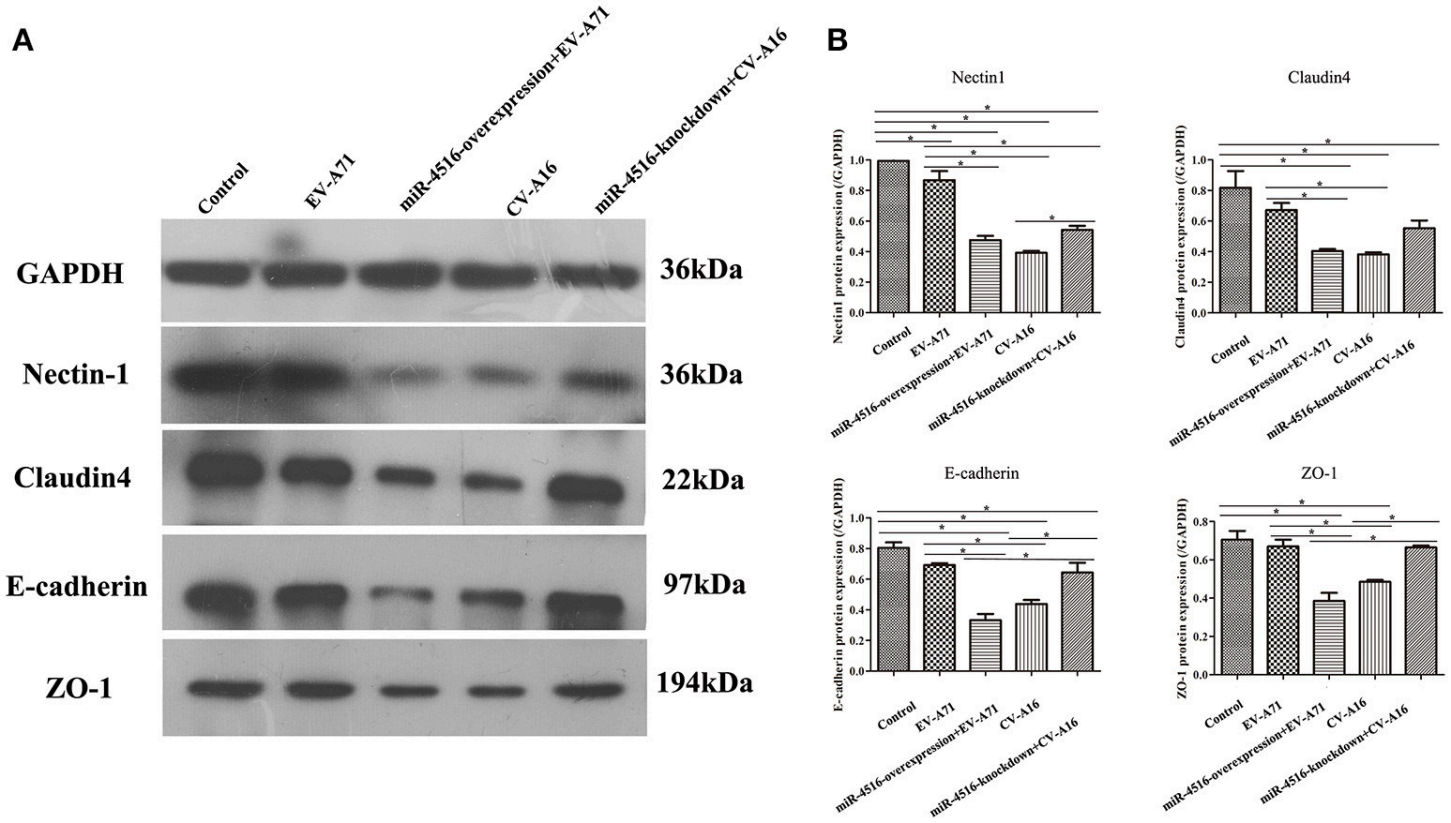
Junctional proteins expression in 16HBE cells infected with EV-A71 or EV-A71 pre-treated with a miR-4516-overexpression plasmid, and CV-A16, or CV-A16 pre-treated with a miR-4516-knockdown plasmid. **(A)** Protein expression of junctional proteins in 16HBE cells with different treatments normalized against GAPDH. **(B)** Densitometry of WBs of three independent experiments performed with lysates at 12 hpi. The signal intensity was normalized to GAPDH levels from the same blots. The results are expressed as the mean± SEM for three subjects. The statistical significance in panel B was assessed with one-way ANOVA followed by a Bonferroni post hoc test. Asterisks indicate statistical significance (^*^P < 0.05).

PVRL1, also known as nectin1, was first identified as an afadin-binding protein and found to serve as a cell adhesion molecule (CAM) at adherens junctions (AJs) (Samanta and Almo, 2015). AJs and TJs are intercellular junctions crucial for the formation and maintenance of the epithelial barrier (Niessen, 2007). Moreover, an increased in cell permeability often results from the disruption of AJ and TJ-related proteins. Therefore, we investigated the role of the opposite expression of miR-4516 in EV-A71 and CV-A16 infections on the distribution of the AJ and TJ-related proteins. At 12 hpi, when an infected monolayer showed significantly higher permeability, the monolayer was stained for AJ and TJ-related proteins and visualized by confocal IF microscopy. First, we investigated the distribution of the direct target of miR-4516. The results are shown in Figure 5A. Cells in the control and si-control groups showed the normal “chicken wire” appearance of the nectin1 structure. Compared to EV-A71-infected cells, which displayed a clear localization of nectin1 at the cell-cell contact sites, nectin1 staining of was markedly lost in CV-A16-infected cells. In contrast, in EV-A71 infected cells that overexpressed miR-4516, most showed a substantial reduction or even the disappearance of the nectin1 staining, while in CV-A16 infected miR-4516-knockdown cells, we observed stronger nectin1 staining at the cell-cell contact sites. Moreover, the other TJ- and AJ-related proteins including claudin4, E-cadherin and ZO-1, (Figures 5B–D) showed a similar alteration as nection1 in each group. Therefore, these observations indicated that EV-A71 and CV-A16 could interfere with AJ and TJ assembly, which was intimately linked to the breakdown of the epithelial barrier, and miR-4516 was differentially expressed following infection with EV-A71 or CV-A16, which might be important regulators involved in the degradation of AJ- and TJ-related proteins. Furthermore, the WB results confirmed these findings (Figure 6). The protein expression levels were slightly and dramatically decreased in EV-A71- and CV-A16-infected cells (*P* < 0.05), respectively, whereas the protein expression levels were remarkably decreased with miR-4516-overexpression pretreatment in an EV-A71 infection and apparently recovered with a miR-4516-knockdown pretreatment in a CV-A16 infection (*P* < 0.05).

## Discussion

Airway epithelial cells are not only the first line of defense against invading microbial pathogens, but also the major route for pathogen entry and the site for the development of infection (Vareille et al., 2011). Accumulating evidence has revealed that airway epithelial cells are also the susceptible cells of EV-A71 and CV-A16 infections. Therefore, in the present study, we adopted 16HBE cells as a cellular model to investigate differences in viral dissemination and pathogenesis in EV-A71 and CV-A16 infections. The results of virus copies and the viral titers indicated that CV-A16 replicated more efficiently than EV-A71 in 16HBE cells, and these observations also correlated well with the WB, IF, and flow cytometry results, showing that the expression level of the VP1 protein and the infection rate in CV-A16 infected-cells was significantly higher than that in EV-A71 infected-cells with time, indirectly suggesting that CV-A16 infected 16HBE cells more easily than EV-A71 did. However, the infectivity of a virus is tightly linked to the occurrence and development of infectious disease. Moreover, studies have reported that the vast majority of patients with impairment of the epithelial lining of the respiratory tract were at greater risk of recurrent respiratory tract infections (Brune et al., 2015). Thus, we speculated whether the greater CV-A16 propagation could cause more serious destruction of the airway epithelial barrier which finally leaded to the recurrent phenomenon of CV-A16 infection. Subsequently, we investigated the effects of EV-A71 and CV-A16 on the permeability of the 16HBE cells and found that the degree of the breakdown of the epithelial barrier in CV-A16 infection was indeed greater than that in EV-A71 infection. Additionally, it was also discovered that apoptosis of 16HBE cells in CV-A16 was more than that in EV-A71 infection. Apoptosis is a critical cellular defense mechanism that contributes to the elimination of pathogen-infected cells in the early phase of an infection, but it can also lead to tissue damage in the later stages of infection and allow virus escape (Ho et al., 2016) For example, dengue virus (DENV) might induce apoptosis that directly causes a breakdown of the blood brain barrier (BBB), allowing it to gain access to the central nervous system (CNS) (Vásquez Ochoa et al., 2009). Moreover, numerous studies have corroborated that apoptosis is the major pathogenic feature of enterovirus infection, ultimately resulting in host cell death and tissue damage (Ho et al., 2016). Therefore, apoptosis caused by EV-A71 or CV-A16 infection in 16HBE cells might partly contribute to the impairments of the epithelial barrier.

Airway epithelial cells serve as a physical barrier mainly because of the structure of intercellular junctions, which are composed of apical TJs and underlying AJs and are linked to the cellular cytoskeleton via numerous adaptor proteins (e.g., ZO proteins; Niessen, 2007). An indispensable role of the intercellular junctions involved in pathogen infection has been widely demonstrated as shown by the dysfunction of the epithelial junction, which leads to an increase in barrier permeability, which in turn may predispose the cell to viral or bacterial infection (Guttman and Finlay, 2009). For example, Escherichia coli directly injects pathogenic effector proteins into the cytoplasm of host cells that collapse localized microvilli, rearrange the cytoskeleton and disrupt intercellular TJs which promote intestinal epithelium permeability, ultimately inducing diarrhea (Muza-Moons et al., 2004; Caron et al., 2006); Adenovirus utilized the coxsackievirus and adenovirus receptor (CAR) protein (a member of JAMs) as a receptor for internalization and to break the epithelial barrier for infection (Walters et al., 2002). Thus, the disruption of these intercellular adhesion-associated molecules might facilitate the destruction of epithelium permeability. Our results further uncovered that some intercellular adhesion-associated molecules in mRNA levels were relatively lower in CV-A16 infection than those in EV-A71 infection, implying that the structure of 16HBE cells subjected to CV-A16 infection might become looser than that in 16HBE cells with EV-A71 infection due to the disturbed junction molecules.

To further excavate the regulators of the disturbed junction molecules during EV-A71 and CV-A16 infections, we focused on miRNAs which not only could regulate the cross-talk between host and pathogens in viral infections, but also is a major component of viral pathogenesis (Ho et al., 2016). In the present study, based on results from our previous study, we screened the miR-4516 and its target PVRL1 (also known as nectin1) which might be the key regulators contributed to the different impairments of the epithelial barrier because of their opposite expression pattern during EV-A71 and CV-A16 infections. It has been reported that high expression levels of miR-4516 were associated with the infiltrative growth of the follicular variant of papillary thyroid carcinomas (Borrelli et al., 2017), but its role in EV-A71 and CV-A16 infections remains unclear. Our WB and IF observations showed that nectin1 was slightly lower and miR-4516 was down-regulated in EV-A71-infected cells, while nectin1 was notably lower and miR-4516 was up-regulated in CV-A16-infected cells, but these alterations of nectin1 were completely reversed in EV-A71- and CV-A16-infected cells pretreated with the miR-4516-up and miR-4516-down plasmids, respectively. Moreover, changes in claudin4, E-cadherin and ZO-1 were similar to those of nectin1 in all groups. Claudin-4 is expressed by epithelial cells as a barrier formed of claudin, and the knockdown of claudin-4 in human distal lung epithelial cells decreased the transepithelial resistance, which indicated the breakdown of the epithelial barrier (Hu et al., 2014); E-cadherin is a typical AJ protein that participates in the establishment of the epithelial cell shape and the maintenance of the differentiated epithelial phenotype (Lecuit and Yap, 2015); ZO-1, a scaffold protein, is essential for TJ formation (Niessen, 2007). These three proteins (i.e., claudin4, E-cadherin and ZO-1) have widely been used as markers of epithelial integrity. Additionally, several studies have demonstrated that nectins might provide the first scaffold for AJ and TJ formation (Niessen, 2007; Samanta and Almo, 2015). Thus, a change in nectins is directly correlated with the intercellular junctions. Collectively, we proposed that differentially expressed miR-4516 induced by EV-A71 and CV-A16 infections might lead to the disruption of cellular junctions by targeting nectin1, which ultimately resulted in perturbation of barrier functions of 16HBE cells.

In conclusion, for the first time, this study demonstrated that miR-4516 regulated the epithelial permeability barrier functions by targeting PVRL1 and might contribute to the differences in the pathogenesis of EV-A71 and CV-A16 infections. It is noteworthy that EV-A71 and CV-A16 both actually broke the epithelial barrier, but comparatively, an opposite miR-4516 expression pattern resulted from EV-A71 and CV-A16 infection in 16HBE cells, which might regulate cell-cell TJs and AJs and consequently cause different degrees of impairment of the epithelial barrier that further facilitate viral infection, especially CV-A16 infection. Thus, these findings might provide a seemingly reasonable explanation as to why CV-A16 infection, but not EV-A71 infection, easily induced a recurrence phenomenon.

## Author contributions

QL and LL conceived and designed the experiments. YH and JS performed the experiments. YH and JS analyzed the data. YH, JS, YZ, and LW contributed reagents, materials, analysis tools. YH and JS wrote the paper. QL and LL edited the manuscript.

### Conflict of interest statement

The authors declare that the research was conducted in the absence of any commercial or financial relationships that could be construed as a potential conflict of interest.

## References

[B1] Aswathyraj S. Arunkumar G. Alidjinou E. K. Hober D. (2016). Hand, foot and mouth disease (HFMD): emerging epidemiology and the need for a vaccine strategy. Med. Microbiol. Immunol. 205, 397–407. 10.1007/s00430-016-0465-y 27406374

[B2] Bhowmik A. Chahal K. Austin G. Chakravorty I. (2009). Improving mucociliary clearance in chronic obstructive pulmonary disease. Respir. Med. 103, 496–502. 10.1016/j.rmed.2008.10.014 19091536

[B3] Boon M. De Boeck K. Jorissen M. Meyts I. (2014). Primary ciliary dyskinesia and humoral immunodeficiency–is there a missing link? Respir. Med. 108, 931–934. 10.1016/j.rmed.2014.03.009 24768622

[B4] Borrelli N. Denaro M. Ugolini C. Poma A. M. Miccoli M. Vitti P.. (2017). miRNA expression profiling of 'noninvasive follicular thyroid neoplasms with papillary-like nuclear features' compared with adenomas and infiltrative follicular variants of papillary thyroid carcinomas. Mod. Pathol. 30, 39–51. 10.1038/modpathol.2016.157 27586203

[B5] Brune K. Frank J. Schwingshackl A. Finigan J. Sidhaye V. K. (2015). Pulmonary epithelial barrier function: some new players and mechanisms. Am. J. Physiol. Lung Cell. Mol. Physiol. 308, L731–L745. 10.1152/ajplung.00309.2014 25637609 PMC4747906

[B6] Cai Y. Ku Z. Liu Q. Leng Q. Huang Z. (2014). A combination vaccine comprising of inactivated enterovirus 71 and coxsackievirus A16 elicits balanced protective immunity against both viruses. Vaccine 32, 2406–2412. 10.1016/j.vaccine.2014.03.012 24657161

[B7] Caron E. Crepin V. F. Simpson N. Knutton S. Garmendia J. Frankel G. (2006). Subversion of actin dynamics by EPEC and EHEC. Curr. Opin. Microbiol. 9, 40–45. 10.1016/j.mib.2005.12.008 16406772

[B8] Guttman J. A. Finlay B. B. (2009). Tight junctions as targets of infectious agents. Biochim. Biophys. Acta 1788, 832–841. 10.1016/j.bbamem.2008.10.028 19059200

[B9] Ho B. C. Yang P. C. Yu S. L. (2016). MicroRNA and Pathogenesis of Enterovirus Infection. Viruses 8:11. 10.3390/v8010011 26751468 PMC4728571

[B10] Hu Q. -P. Sun Q. He N. Fu Y.-F. Zhu W.-P. Song H.-H. (2014). Epidemiological analysis of single and repeated infections with hand-foot-mouth disease in Pudong New Area of Shanghai City. Chin. J. Disease Control Prevent. 18, 363–365.

[B11] Hu Y. Song J. Liu L. Li J. Tang B. Zhang Y.. (2017). Comparison analysis of microRNAs in response to EV71 and CA16 infection in human bronchial epithelial cells by high-throughput sequencing to reveal differential infective mechanisms. Virus Res. 228, 90–101. 10.1016/j.virusres.2016.11.024 27890633

[B12] Koyama A. H. Adachi A. Irie H. (2003). Physiological significance of apoptosis during animal virus infection. Int. Rev. Immunol. 22, 341–359. 10.1080/08830180305210 12959749

[B13] Lecuit T. Yap A. S. (2015). E-cadherin junctions as active mechanical integrators in tissue dynamics. Nat. Cell Biol. 17, 533–539. 10.1038/ncb3136 25925582

[B14] Lee J. J. Seah J. B. Chow V. T. Poh C. L. Tan E. L. (2011). Comparative proteome analyses of host protein expression in response to Enterovirus 71 and Coxsackievirus A16 infections. J. Proteomics 74, 2018–2024. 10.1016/j.jprot.2011.05.022 21621020

[B15] Lin J. Y. Shih S. R. (2014). Cell and tissue tropism of enterovirus 71 and other enteroviruses infections. J. Biomed. Sci. 21:18. 10.1186/1423-0127-21-18 24602216 PMC3995930

[B16] Livraghi A. Randell S. H. (2007). Cystic fibrosis and other respiratory diseases of impaired mucus clearance. Toxicol. Pathol. 35, 116–129. 10.1080/01926230601060025 17325980

[B17] Lui Y. L. Timms P. Hafner L. M. Tan T. L. Tan K. H. Tan E. L. (2013). Characterisation of enterovirus 71 replication kinetics in human colorectal cell line, HT29. Springerplus 2:267. 10.1186/2193-1801-2-267 23875129 PMC3696168

[B18] Mateo M. Generous A. Sinn P. L. Cattaneo R. (2015). Connections matter–how viruses use cell-cell adhesion components. J. Cell Sci. 128, 431–439. 10.1242/jcs.159400 26046138 PMC4311127

[B19] Muza-Moons M. M. Schneeberger E. E. Hecht G. A. (2004). Enteropathogenic Escherichia coli infection leads to appearance of aberrant tight junctions strands in the lateral membrane of intestinal epithelial cells. Cell. Microbiol. 6, 783–793. 10.1111/j.1462-5822.2004.00404.x 15236645

[B20] Niessen C. M. (2007). Tight junctions/adherens junctions: basic structure and function. J. Invest. Dermatol. 127, 2525–2532. 10.1038/sj.jid.5700865 17934504

[B21] Sajjan U. Wang Q. Zhao Y. Gruenert D. C. Hershenson M. B. (2008). Rhinovirus disrupts the barrier function of polarized airway epithelial cells. Am. J. Respir. Crit. Care Med. 178, 1271–1281. 10.1164/rccm.200801-136OC 18787220 PMC2599868

[B22] Samanta D. Almo S. C. (2015). Nectin family of cell-adhesion molecules: structural and molecular aspects of function and specificity. Cell. Mol. Life Sci. 72, 645–658. 10.1007/s00018-014-1763-4 25326769 PMC11113404

[B23] Sun S. Jiang L. Liang Z. Mao Q. Su W. Zhang H.. (2014). Evaluation of monovalent and bivalent vaccines against lethal Enterovirus 71 and Coxsackievirus A16 infection in newborn mice. Hum. Vaccin. Immunother. 10, 2885–2895. 10.4161/hv.29823 25483672 PMC5443096

[B24] Vareille M. Kieninger E. Edwards M. R. Regamey N. (2011). The airway epithelium: soldier in the fight against respiratory viruses. Clin. Microbiol. Rev. 24, 210–229. 10.1128/CMR.00014-10 21233513 PMC3021210

[B25] Vásquez Ochoa M. Garcia Cordero J. Gutierrez Castaneda B. Santos Argumedo L. Villegas Sepulveda N. Cedillo Barron L. (2009). A clinical isolate of dengue virus and its proteins induce apoptosis in HMEC-1 cells: a possible implication in pathogenesis. Arch. Virol. 154, 919–928. 10.1007/s00705-009-0396-7 19440830

[B26] Walters R. W. Freimuth P. Moninger T. O. Ganske I. Zabner J. Welsh M. J. (2002). Adenovirus fiber disrupts CAR-mediated intercellular adhesion allowing virus escape. Cell 110, 789–799. 10.1016/S0092-8674(02)00912-1 12297051

[B27] Wang J. F. Guo Y. S. Christakos G. Yang W. Z. Liao Y. L. Li Z. J.. (2011). Hand, foot and mouth disease: spatiotemporal transmission and climate. Int. J. Health Geogr. 10:25. 10.1186/1476-072X-10-25 21466689 PMC3079592

[B28] Wang T. Shuai H. Deng A. Fang Y. Zhou Q. Sun L. (2013). Characters of repeated infection of Hand-Foot-Mouth disease. Guide China Med. 11, 38–40. 10.15912/j.cnki.gocm.2013.13.504

[B29] Zhao T. Zhang Z. Zhang Y. Feng M. Fan S. Wang L.. (2017). Dynamic interaction of enterovirus 71 and dendritic cells in infected neonatal Rhesus Macaques. Front. Cell. Infect. Microbiol. 7:171. 10.3389/fcimb.2017.00171 28540257 PMC5423916

