# Supplementary material for: microRNA-4516 Contributes to Different Functions of Epithelial Permeability Barrier by Targeting Poliovirus Receptor Related Protein 1 in Enterovirus 71 and Coxsackievirus A16 Infections
Source: Front Cell Infect Microbiol. 2018 Apr 9;8:110. doi: 10.3389/fcimb.2018.00110 (PMC5900049; doi:10.3389/fcimb.2018.00110)
Supplement: Supplementary file 1 [file Table1.DOCX]

**Supplementary Material:**

**Table S1. Details of primers and probes used in the study.**

| **Genes or miRNAs** | **Primers and probes sequences** | **Application** |
| --- | --- | --- |
| EV-A71-VP1-1 | 5’-TGCCAACTGGGACATAGATATAACAGG-3’ (sense)  5’-ACTCTAAAGTTGCCCACATAAATAGCC-3’(anti-sense) | Construction of standard RNA |
| EV-A71-VP1-2 | 5’-ACCTATTCAAAGCCAACCCAA-3’ (sense)  5’-TAAATAGCCCCGGACTGTTGT-3’(anti-sense)  Probe: FAM-TTTCCCAAGAGTGGTGATCGCTGT-TAMRA | qRT-PCR |
| CV-A16-VP1-1 | 5’-AACACTGAGGCTAGTAGTCAC-3’ (sense)  5’-CGTGTTTGATTCTCATGTACACC-3’(anti-sense) | Construction of standard RNA |
| CV-A16-VP1-2 | 5’-GTTTGTGAAAATGACGGACCC-3’ (sense)  5’-GTCATTTGCTTGAAGGTGCTC-3’(anti-sense)  Probe: FAM-CAGCTCAAGTGTCAGTCCCCT-TAMRA | qRT-PCR |
| GAPDH | 5’- AGAAGGCTGGGGCTCATTTG -3’ (sense)  5’-AGGGGCCATCCACAGTCTTC-3’(anti-sense) | qRT-PCR |
| ICAM-1 | 5’-CCTCCTGTGACCAGCCCATCTCGTT-3’ (sense)  5’-CCCACCCTCCACCTGGCAGCGTA-3’(anti-sense) | qRT-PCR |
| VCAM-1 | 5’-GTGACTCCGTCTCATTGACTTGC-3’ (sense)  5’-AACTAACAGGATTCATTGTCAGCGTA-3’(anti-sense) | qRT-PCR |
| E-cadherin | 5’-AGGCAAGGTTTTCTACAGCATCACT-3’ (sense)  5’-ATGTGGCAATGCGTTCTCTATCCAG-3’(anti-sense) | qRT-PCR |
| occludin | 5’-TTCTCTTGATGAGCACCTTTAACTGA-3’ (sense)  5’-TACTGACAAACTTGCCTTCACGAAC-3’(anti-sense) | qRT-PCR |
| Connexin | 5’-TCCCGACGCAGAGCAAACC-3’ (sense)  5’-TTTGCAGCCACAACGAGGAT-3’(anti-sense) | qRT-PCR |
| claudin-5 | 5’-AGATTGAGAGGTCTGGGAAGC-3’ (sense)  5’-TCTCTCATCCCATGGCAAACA-3’(anti-sense) |  |
| claudin4 | 5’-TCTCCTCTGTTCCGGGTAGG-3’ (sense)  5’-CGTCCATCCACTCTGCACTT-3’(anti-sense) | qRT-PCR |
| claudin18 | 5’-GTGGCGTTCCTCCTGTCCAT-3’ (sense)  5’-TCCAGAGCCCTTCGTACTGG-3’(anti-sense) | qRT-PCR |
| ZO-1 | 5’-CCAGCCTGCTAAACCTACTAAAGTCAC-3’ (sense)  5’-GGACATTCAATAGCGTAGCCCGTTC-3’(anti-sense) | qRT-PCR |
| miR-4516 | 5’-ACACTCCAGCTGGGGGGAGAAGGGT-3’ (sense) | qRT-PCR |
| PVRL1 | 5’-CTTGGCCTGCATCGTCAACTACCACA-3’ (sense)  5’-GCAGGTACCAGTTGCCATCAAACCC-3’(anti-sense) | qRT-PCR |
